# The role of ATP-binding cassette transporter A2 in childhood acute lymphoblastic leukemia multidrug resistance

**Published:** 2014-07-20

**Authors:** N Aberuyi, S Rahgozar, A Moafi

**Affiliations:** 1 Division of Cell and Molecular Biology, Department of Biology, Faculty of Science, University of Isfahan, Iran; 2 Department of Paediatric-Oncology, SayedolShohada Hospital, Isfahan University of Medical Sciences, Isfahan, Iran

**Keywords:** Leukemia, drug resistance, ATP-binding cassette transporter

## Abstract

Acute lymphoblastic leukemia (ALL) is one of the most prevalent hematologic malignancies in children. Although the cure rate of ALL has improved over the past decades, the most important reason for ALL treatment failure is multidrug resistance (MDR) phenomenon. The current study aims to explain the mechanisms involved in multidrug resistance of childhood ALL, and introduces ATP-binding cassette transporterA2 (ABCA2) as an ABC transporter gene which may have a high impact on MDR.

Benefiting from articles published inreputable journals from1994 to date and experiments newly performed by our group, a comprehensive review is written about ABCA2 and its role in MDR regarding childhood ALL.

ABCA2 transports drugs from the cytoplasm into the lysosomal compartment, where they may become degraded and exported from the cell. The aforementioned mechanism may contribute to MDR. It has been reported that ABCA2 may induce resistance to mitoxantrone, estrogen derivatives and estramustine. It is resistant to the aforementioned compounds. Furthermore, the overexpression ofABCA2 in methotrexate, vinblastine and/or doxorubicin treated Jurkat cells are observed in several publications. The recent study of our group showsthatthe overexpression ofABCA2 gene in children with ALL increases the risk of MDR by 15 times.

ABCA2 is the second identified member of the ABCA; ABC transporters' subfamily. ABCA2 gene expression profile is suggested to be an unfavorable prognostic factor in ALL treatment. Better understanding of the MDR mechanisms and the factors involved may improve the therapeutic outcome of ALL by modifying the treatment protocols.

## Introduction

Cancer is the second most important factor causing death in developing countries (1) and a main health concern worldwide (2). Leukemia is one of the commonly reported cancers (9%) (3) including34% of all cases among the children under15-year-olds (4) and 28to30% of cases below 18-year-olds (5).Acute lymphoblastic leukemia (ALL)is one of the four types of hematologic malignancies among children with the highest frequency (6).Despite enhancing the efficiency of treatment for childhood ALL, multi drug resistance (MDR) is remained a serious impediment in this regard (7-11). MDR is a multifactorial phenomenon (12, 13) which mostly attributed to ABC transporters (10, 12, 13). ABCA2 is the second member of the A subfamily of ABC transporters which may have a role in MDR (14, 15).Our group has recently suggested that ABCA2is related to poor prognosis in childhood ALL (7, 16, 17).The present study is a literature based review article which explains the aforementioned topics, especially the impact of ABCA2 in multidrug resistance of childhood ALL.

**Acute lymphoblastic leukemia (ALL)**

Acute lymphoblastic leukemia is one of the most important blood cancers in human kind (6) and it is a malignant illness of white blood cells (18). This kind of leukemia includes 32 percent of cancers in children under 15 years old (19) and it includes 80 percent of cases of blood cancers in children (5, 7, 8). The vast majority of the cases are between 2 to 5 years old (20). In a research that is conducted in Iran, it is revealed that this prevalent cancer contains 36 percent of the cases (21). The World Health Organization (WHO) divides ALL into 3 groups (22):

1.Precursor B acute lymphoblastic leukemia/lymphoma

2.Burkitt leukemia/lymphoma/mature B

3.Precursor T acute lymphoblastic leukemia/lymphoma

Genetic and environmental factors engage in accession of ALL. The examples of these genetic factors are wrong expression of protoancogenes, more than 50 chromosome hyperdiploidy and chromosome translocations that create fusion genes that code active kinases and changed transcription factors (23, 24). These disorders maybe influenced by changed DNA repair and the control processes of cell cycle(25). The instances of influential environmental factors are the impact of electromagnetic waves, some viral pollutions and smoking of parents.These factors help the progress of leukemia by making secondary genetic changes. Some rare genetic syndromes exposed under radio therapy, and heavyweight born children are instances of some other factors which may improve the risk of ALL.The rate of survival in childhood ALL had a significant increase in some countries, during more than four decades (26). Five-year survival rates, since 1990 to 1994 raised to 83.7% (27). Some countries have achievedthe rate of long-term survival, in more than 80% of children suffering from ALL from 1 to 10 years old (28, 29). Including them, Iran has revealed a survival rate of 56.6% in a study performed in Shiraz (30).However, despite the successes in treatment of childhood ALL, the difficult challenge in the treatment of these children is resistance to chemotherapy (7-11). Due to the increased outflow of a wide range of chemotherapeutic drugs from inside of the cell to the outside, this resistance was named multidrug resistance (10). In ALL, the biological features of leukemia cells (number and translocations of chromosomes) and clinical properties (including: age, leukocyte counting at the time of diagnosis) are efficient prognostic factors for treatment selectionincluding the number and dosage of drugs chosen in chemotherapy. 

**Multidrug resistance (MDR)**

**Figure 1:**
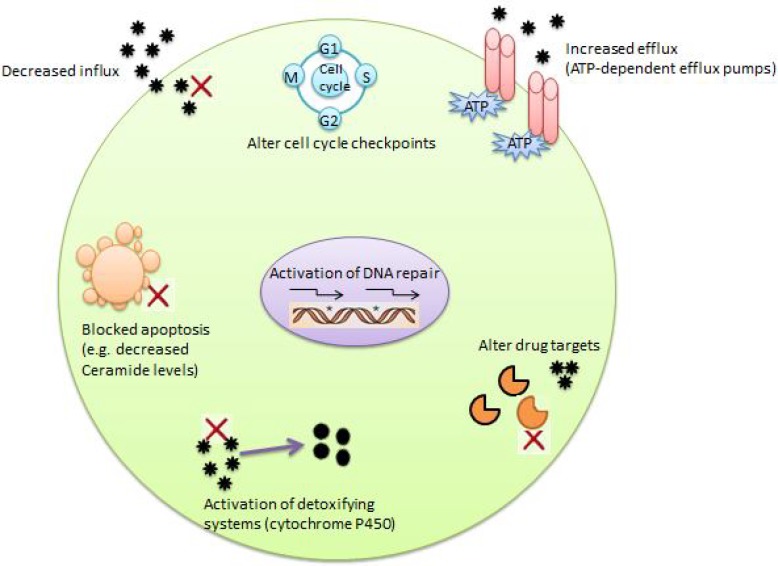
Several mechanisms of MDR in cancer cells.MDR can occur in cancer cells bymechanisms including (a) decreased influx of drug, (b) alterationof cell cycle checkpoints, (c) increased efflux of drugs by ATP-dependent pumps, (d) blocked apoptosis, (e) increased DNA repair, (f) altered drug targets and (g) activation of detoxificationsystems

MDR is a phenomenonthrough which cancer cells becomeresistant to variety of anti-cancer drugs which are functionally and structurally unrelated and discrete (10, 12, 13, 31, 32). MDR mechanisms in cancers were studied extensivelyand identified as multifactorial and complex phenomenon (12, 13). MDR induction is related to the changes of some molecular pathways (33). The loss of drug transporter proteins on the surface of the cell, change or mutation in drug specific targets (12), decrease in absorption of water soluble drugs, DNA damage repair, decrease in apoptosis and increase in energy dependent efflux of hydrophobic drugs affect these molecular pathways (33, 34)(figure 1).

There are 2 groups of "resistance to anti-cancer drugs":

1. Those that disturb drug delivery to cancer cells.

2. Those that emerge in cancer cells through genetic and epigenetic changes and affect drug sensitivity.

**Figure 2:**
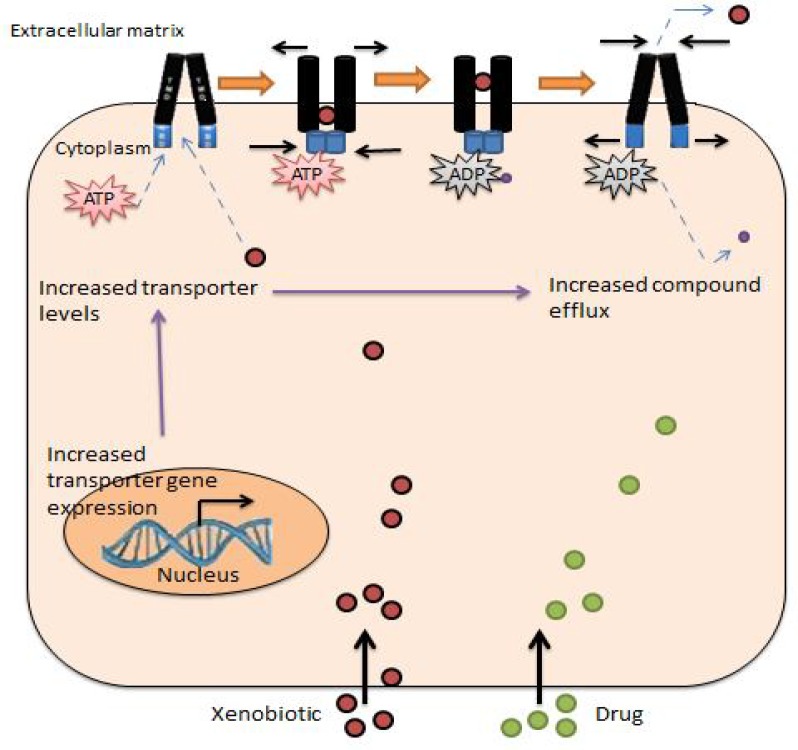
Function ofABC transporters in MDR and their drug efflux mechanism. The entry ofdruginto the cell by diffusion or active uptake leads to increased transporter gene expression, increased transporter levels and so increased compound efflux.After substrate and ATP binding to ATP-dependent pumps, thetransporter effluxessubstrate to extracellular matrix. In this mechanism, Phosphate group is released, and the substrate is then excreted

Dysfunction in drug delivery can be the result of weak absorption of prescribed oral medicines or increase in medicinal metabolism and excretion, which may contribute to tumor growth (35). A proved and important recently proposedreason of MDR (10, 12, 13, 33, 36, 37) (figure 2) is the increased expression of ATP-binding cassette (ABC) transporters, that extract a variety of chemotherapeutical drugs from the cell and prevent an appropriate response to cure (10, 38, 39).

**ABCA2 transporter**

ABC transporters are transmembrane proteins[40]acting as auniportcarrier (to inside or outside of the cell) of a variety of substrates (36, 41-44) across the intracellular (organelles) and cytoplasmic membrane (33, 40, 45). Thisunitransport is against theconcentration gradient and depends on ATP hydrolysis (33, 34, 44, 46). This large group of protein family which has 49 members (33, 40, 42, 47-49) is divided into 7 subfamilies from A to G (12, 36, 40, 44, 46-48, 50-52). These transporters are engaged in energy dependent transporting of xenobiotic and other toxic compounds, as well as, anti-cancer natural materials (12),and consequently they develop resistance to chemotherapy in various cancers including lymphoblastic and myeloid leukemia (53).

**Figure 3:**
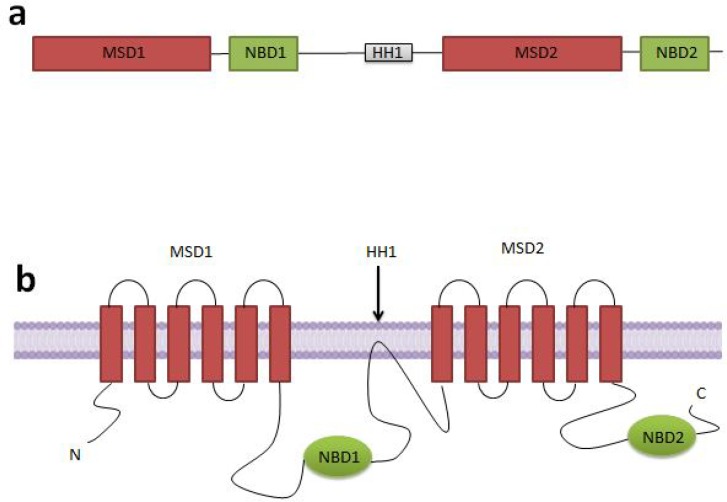
Gene (a) and protein (b) structure of ABCA2 transporter. There is a long cytoplasmic regulator domain between two symmetric halves of this protein that each of them contains a membrane including a spanning domain(MSD) and a nucleotide binding domain(NBD).

The second member of ABCA subfamily is anendolysosomic protein which is related to lipid transport and drug resistance and is named ABCA2 (54, 55). It is a large and symmetricproteinwith a channel-like functional structure. This functional structure is placed in the membrane (56). ABCA2 protein, like other members of this family, contains two symmetric halvescontaining a long cytoplasmic regulator domain in between. Cytoplasmic regulator domain contains a high hydrophobic sequence that dips into the membrane (figure 3) (14). This protein contains 2436 amino acids (43, 57) and its molecular weight is almost 250 kDa (14, 57). ABCA2 protein has the highest homology with ABCA1(14, 57-59) and it is cleared that this high amount of same homology is due to the duplication of ancestral gene during the evolution[14]. This gene is located on the q arm of chromosome 9, near the ABCA1 gene (13, 14, 42, 43, 47, 56, 57, 59-61). ABCA2 gene contains 48 exones (54) and in comparison with other transporters that have 32to250 kbp gene length,but this gene, unexpectedly, has 21 kbp (14, 57).The coding regionlength of this gene is 7.3 kbp (57). Additionally, two transcriptions are recognized for this gene(62). ABCA2 protein, is located on inner vesicles (63) such as late endosomes, lysosomes (14, 64-66), trans Golgi (67) and endoplasmic reticulum (44, 45, 55, 62, 68, 69). For detoxification, ABCA2 transporter protein imports waste output of the cell and toxic compounds from cytoplasm to lysosomes (47, 69, 70).

ABCA2 transports a variety of substrates (10, 35, 51, 66, 67, 71, 72) that they have commonality with toxic compounds of ABCA3 substrates (38). ABCA2 is the third unknown member of ABC transporters subfamilyand it has a role in occurrence of MDR phenotype in childhood ALL (16).

**Figure 4:**
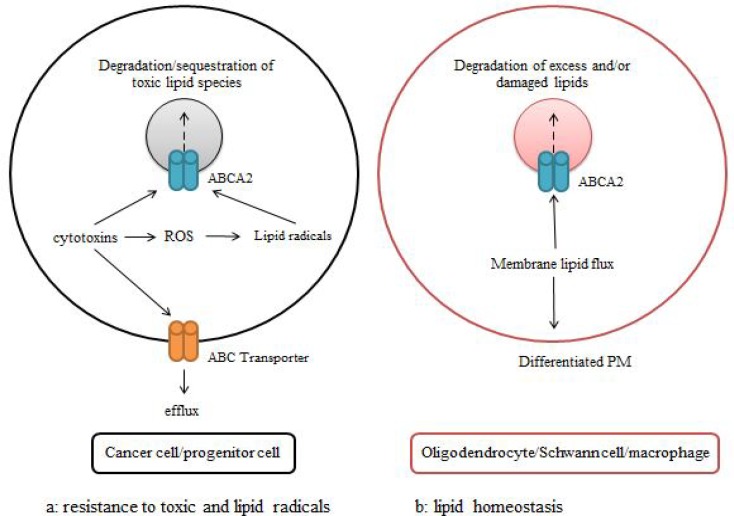
The function of ABCA2 protein. a:trapping of toxic lipids and/or lipid radicals in the cancer and progenitor cells for protecting the celland degradation of toxic substance. b: contributing to the degradation of membrane lipids in cell differentiation to maintain lipid homeostasis

ABCA2, is naturally expressed in some tissues, such as, blood cells (15), including macrophages (73, 74), monocytes (35, 42) and blood stem cells (50, 51).Its important rolein some of these cells including macrophages and neurons is lipidhomeostasisand metabolism(figure 4) (14, 75).ABCA2 is also expressed in cancer cell lines (43).It is believedthat during tumor growth, the gene expressionincreasesinorder tomaintain tumor cellproliferation (15).ABCA2gene promoterhas a cholesterol- response element [76]and cholesterol has a role in regulating thesurvivaland differentiation ofnormal and tumor cells. Therefore, 

it can be presumed that ABCA2mayalsoplay a role incancer development (15).Gene dysfunction causes latency in metastasis and chemotactic migration in prostate cancer (74). ABCA2 protein is related to diseases such as, early atherosclerosis (46, 67), Tangier's, small cell lung cancer, acute myeloid leukemia (71) and Alzheimer's disease early infection (46, 67, 71, 77). This transporter protein may cause drug resistance in response to cancer chemotherapy (13).

**ABCA2 and MDR in ALL**

MDR1 is one of the drug resistance genes that produces P-glycoprotein (p-gp). This gene is expressed in some cancers. However, in ALL, there is a controversy regarding the relationship between p-gp and MDR (7, 38). Although, some researchers reported that the high expression of this protein and its function is related to the failure of all chemotherapy and poor prognosis (78), some others did not observe such a relationship (38, 79). It is probable that these contradictory results are due to different experimental methods used in these studies. The role of ABCA2 protein in drug transport and its role in MDR are under investigation. It is suggested that this transporter has a role in MDR by saving drugs in the lysosome and probably their efflux from the cell (figure 4) (14, 15, 80). Although, there are few reports that show the in vivo impact of ABCA2 in MDR, considerable number of experimental studies have revealed this relationship (73). For instance, drug resistance to metoxantrone(14, 59, 70, 81), Estrogen derivatives (42) and Stramostin (14, 42, 43, 59, 70, 74) are observed in cell lines with high expression of ABCA2(35). Stramostin is an anti-microtubule drug which is used for chemotherapy of ovarian and prostate cancers. In comparison with parental cell line, prostate and ovarian cancer cell lines, resistant to Stramostin,demonstrate more expression of ABCA2 in mRNA (41, 42, 55) and protein (74) levels. High protein expression levels of ABCA2 are reported in human malignant mesothelioma (MM) cell lines resistant to doxorubicin.This overexpression may decrease drug centralization through activating "extra cellular signal regulated kinases", named ERK1 and ERK2 which may contribute to tumor growth (82). Some of the studies showed that the level of ABCA2 protein in patients having chronic myeloid leukemia has no relationship with response to imatinib drug (51),while, some otherstudieshave shown that ABCA2 protein leads to drug resistancein T-ALL and AML (83). In 2006, Steinbach and his colleagues studiedthe mRNA expression profiles of 38 ABC transporter genes in patients with AML in 25, 50 and 75 age levelsdivided into two groups with good and poor response to chemotherapy. It was demonstrated that patients with high mRNA levels of ABCA2, besides three other studied genes, were included in the group of patients with poor response to chemotherapy (53).Gillet and colleagues identified ABCA2 as one of the genes which was highly expressed in multidrug resistant cell lines of breast cancer( MCF7/CH1000), AML (HL60/AR) and T-ALL(CEM/ADR5000)[13]. Treatment of acute lymphoblastic leukemia cell lines of CCRF-CEM and Jurkat with methotrexate, vinblastine or doxorubicin caused certain increase of ABCA2 expression in Jurkat cell line, and of ABCA3 in CCRE-CEM and Jurkat cell lines (38, 70). Blocking of ABCA2 and ABCA3 genes by siRNAs showed drug sensitivity in the aforementioned cell lines[38].A reparative mechanism is proposed relating ABCA3 to ABCA2 transporter, where the enhanced activity of one protein may compensate the dysfunction of the other one(70). In a recent studyperformed by our group, it is revealed that four ABC transporters are related to poor prognosis in childhood ALL (7, 17). Among them ABCA2 high mRNA levels increase the risk of MDR 15 fold more than those with lower expressions (16).

The regulation of gene expression can occur at various levels including transcriptional, post-transcriptional and post-translational levels. The post-translational modifications may include alterations in splicing control, mRNA consistency, localization and translation[84]. For example, it is shown that in some patients with lung cancer,despite the increased mRNA levels of Mdr1 gene, no enhanced expression at protein levels was observed(85). Further investigations are required to delineate the mechanisms through which ABCA2 may influence multidrug resistance in childhood ALL.

**Conclusion**:

ALL isthe most prevalent cancer in children under 15 years old.Multidrug resistance is one of the most important obstacles of treatment in these patients. The current review article has discussed the possible role of ABC transporter, ABCA2, in MDR and the poor prognostic value of this gene in childhood ALL according to several, recent published studies in this field. It is hoped that this manuscript would help for a better understanding of this gene and its impact on multidrug resistance; the information which may improveour knowledge for choosing more effective protocols for treating acute lymphoblastic leukemia.
